# Supplementary material for: NLRP3 phosphorylation in its LRR domain critically regulates inflammasome assembly
Source: Nat Commun. 2021 Oct 6;12:5862. doi: 10.1038/s41467-021-26142-w (PMC8494922; doi:10.1038/s41467-021-26142-w)
Supplement: Supplementary file 1 — Supplementary Information [file 41467_2021_26142_MOESM1_ESM.pdf]

NLRP3 phosphorylation in its LRR domain critically regulates inflammasome assembly.

Tingting Niu[1,2], Charlotte De Rosny[1], Séverine Chautard[1], Amaury Rey[1], Danish Patoli[1], Marine Groslambert[1], Camille Cosson[1], Brice Lagrange[1], Zhirong Zhang[3], Orane Visvikis[4], Sabine Hacot[5], Maggy Hologne[6], Olivier Walker[6], Jeimin Wong[2], Ping Wang[7], Roméo Ricci[3], Thomas Henry[1], Laurent Boyer[4], Virginie Petrilli[5], and Bénédicte F. Py [1,*]

Supplementary Information

Supplementary Figs. 1-10

Supplementary Tables 1-5

Supplementary Figure 1

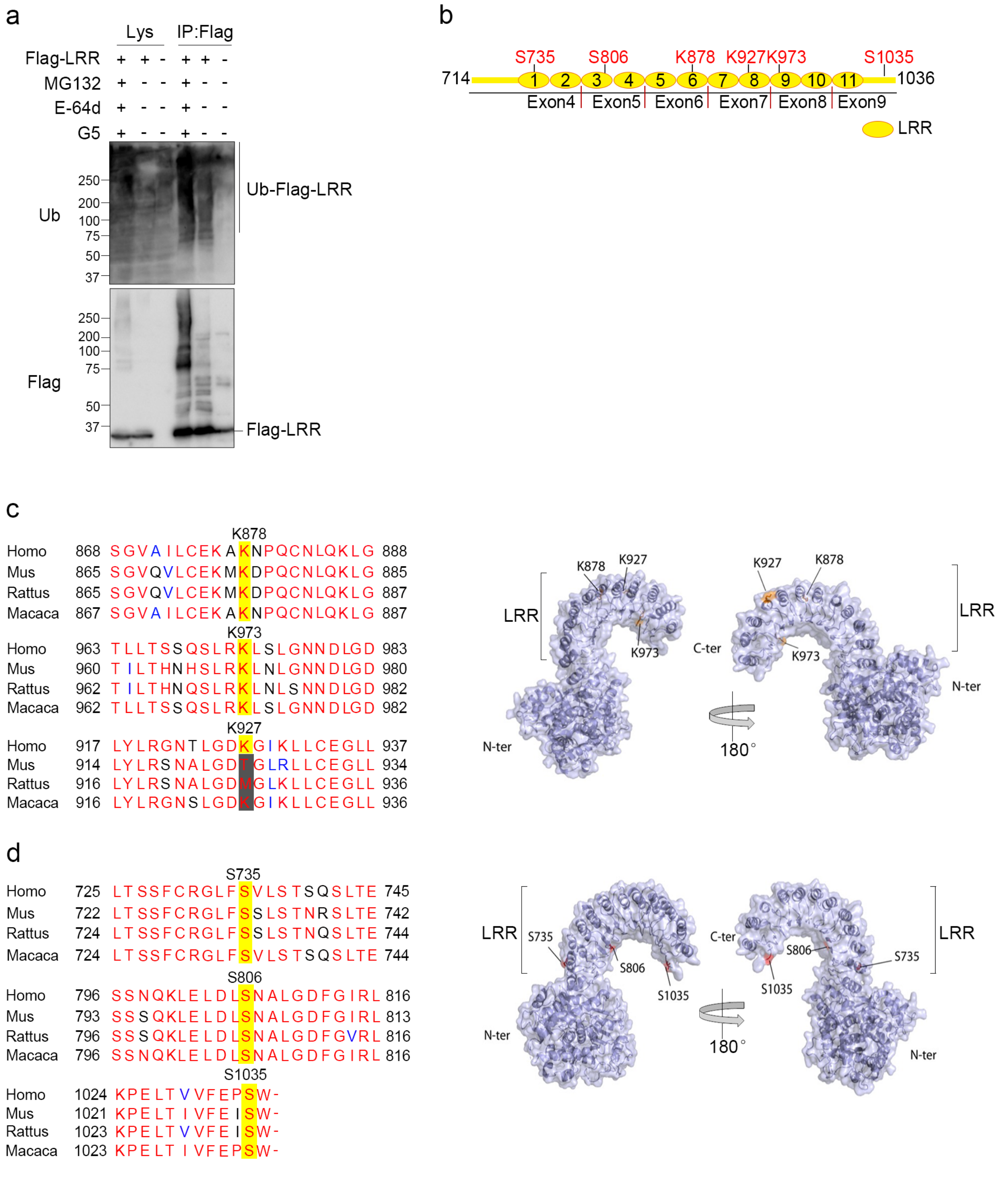

Supplementary Fig. 1. NLRP3 is ubiquitinated at K878, K927, K973, and phosphorylated at S735, S806, S1035. **a** 293T cells were transfected with plasmids coding for Flag-LRR. 1 day later, cells were treated with MG132 (10 μM), E-64d (20 μg/ml), and G5 (1 μM) for 30 min. Flag-LRR was purified by anti-Flag immunoprecipitation and analyzed by SDS-PAGE followed by anti-Flag and anti-Ub WB. **b** Mapping of the identified modified residues in NLRP3 LRR domain structural and genomic sequences. Exons are numbered according to Exon1 encoding the ATG start codon. **c** K878 and K973 are conserved among species, but K927 is specific to human NLRP3. Alignments were performed using ClustalW2. Surface representation of the NACHT+LRR domains model of NLRP3 (6NPY.PDB) using PyMOL.[16] K878, K927, K973 (represented in orange) are all exposed at the surface of the NLRP3 LRR domain. **d** S735, S806 and S1035 are conserved among species and exposed at the surface of NLRP3 LRR domain. S735, S806 and S1035 are represented in red in the surface representation of the NACHT+LRR domains model of NLRP3 (6NPY.PDB) using PyMOL.[16] Data correspond to the one selected experiment used to perform the mass spectrometry analysis, out of 8 independent repeats. Molecular weights are indicated in kDa (a). Lys, lysates; IP: Flag, anti-Flag immunoprecipitates; Ub, ubiquitin.

Supplementary Figure 2

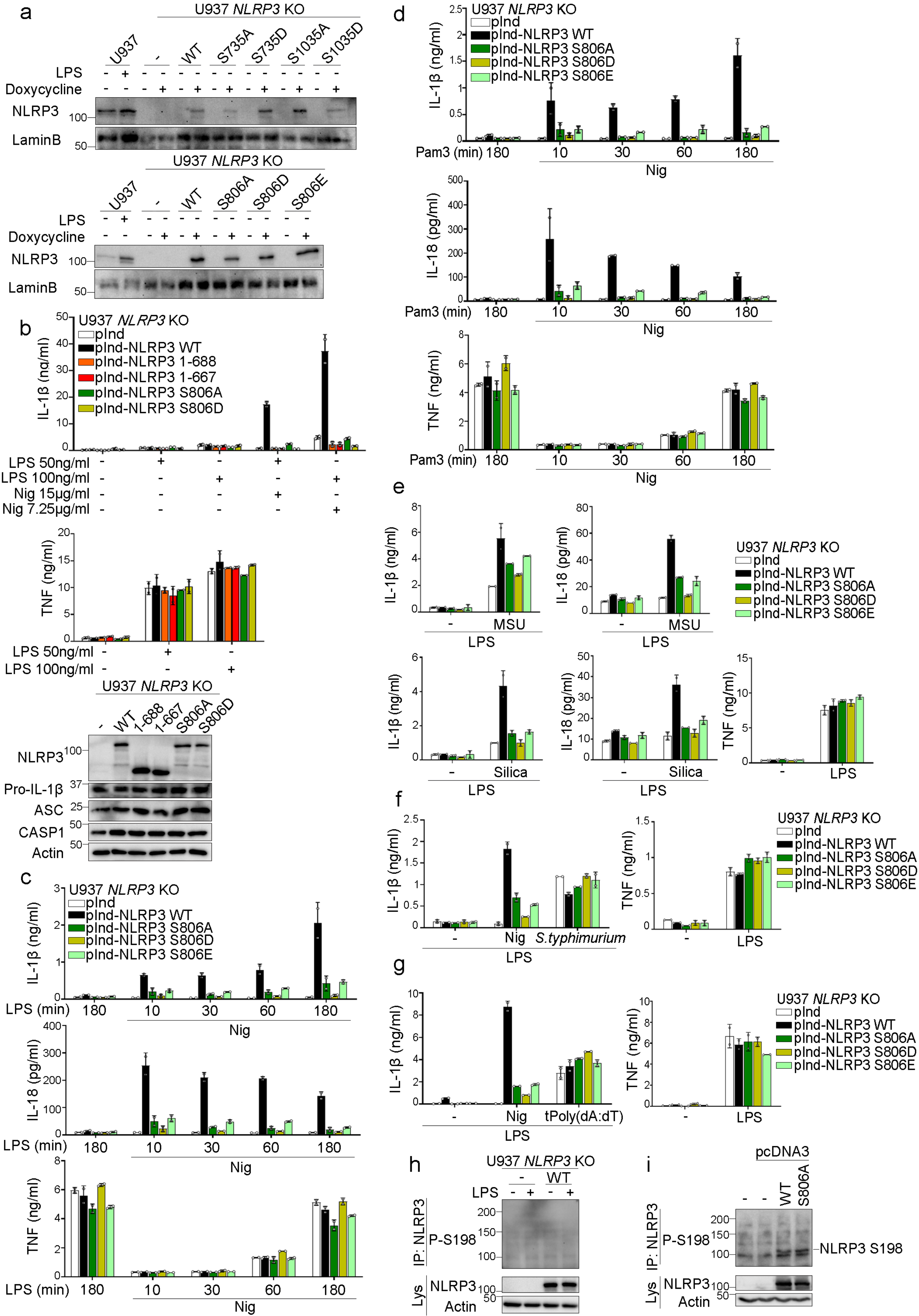

Supplementary Fig. 2. S806 is critical for NLRP3 inflammasome activity in human U937 monocytes. **a** U937 and NLRP3-deficient U937 cells reconstituted with NLRP3 mutants were differentiated with PMA and treated with doxycycline (2 μg/ml, 4h) or LPS (50 ng/ml, 4h) as indicated. NLRP3-deficient U937 cells reconstituted with empty lentivector were used as controls (-). NLRP3 expression was analyzed by WB. **b** NLRP3-deficient U937 cells reconstituted with NLRP3 mutants were differentiated with PMA and treated with doxycycline (2 μg/ml) and then with LPS (50 ng/ml, 4h) followed by nigericin (15 μg/ml, 1h), or PMA (50 ng/ml) for 16h, and then doxycycline (2 μg/ml) and LPS (100 ng/ml) for 11h, before media change and addition of nigericin (7.25 μg/ml, 1h). NLRP3-deficient U937 cells reconstituted with empty lentivector were used as controls (-). NLRP3 expression was analyzed by WB. **c** NLRP3-deficient U937 cells reconstituted with NLRP3 mutants were differentiated with PMA and treated with doxycycline (2 μg/ml, 4h) and LPS (50 ng/ml, time as indicated) followed by nigericin (15 μg/ml, 1h). IL-1β, IL-18 and TNF secretions were measured by ELISA. **d** NLRP3-deficient U937 cells reconstituted with doxycycline-inducible NLRP3 mutants were differentiated with PMA and treated with doxycycline (2 μg/ml, 4h) and Pam3CSK4 (1 μg/ml, time as indicated) followed by nigericin (15 μg/ml, 1h). IL-1β, IL-18 and TNF secretions were measured by ELISA. **e** NLRP3-deficient U937 cells reconstituted with NLRP3 mutants were sequentially treated with PMA, doxycycline (2 μg/ml, 4h), LPS (50 ng/ml, 4h) and MSU (250 μg/ml, 16h) or silica (25 μg/ml, 16h). IL-1β, IL-18 and TNF secretions were measured by ELISA. **f** NLRP3-deficient U937 cells reconstituted with NLRP3 mutants were sequentially treated with PMA, doxycycline (2 μg/ml, 4h), LPS (50

ng/ml, 3h) and infected with *S. Typhimurium* SL1344 (MOI 2.4, 2h). IL-1β and TNF secretions were measured by ELISA. **g** NLRP3-deficient U937 cells reconstituted with NLRP3 mutants were treated with PMA, doxycycline (2 μg/ml, 4h), LPS (50 ng/ml, 3h) and transfected with poly(dA:dT) (1 μg/ml, 6h). IL-1β and TNF secretions were measured by ELISA. **h** NLRP3-deficient U937 cells reconstituted with doxycycline-inducible NLRP3 were treated with PMA (50 ng/ml) and doxycycline (2 μg/ml) for 16h, and then with LPS (50 ng/ml, 6h). Anti-NLRP3 immunoprecipitates were analyzed for $pNLRP3_{S198}$ using phospho-specific human $pNLRP3_{S198}$ antibodies by WB. **i** 293T cells ectopically expressing human NLRP3 WT or S806D mutant were analyzed by anti-NLRP3 immunoprecipitation followed by anti-$pNLRP3_{S198}$ WB. Means and 1 SD are shown. Data are representative of 2 independent experiments (a, b, h, i) and biological duplicates representative of 2 (f, g) and 3 (b, c, d, e) independent experiments. Molecular weights are indicated in kDa (a, b, h, i). pInd, pInducer21; Lys, lysates; IP: NLRP3, anti-NLRP3 immunoprecipitates.

Supplementary Figure 3

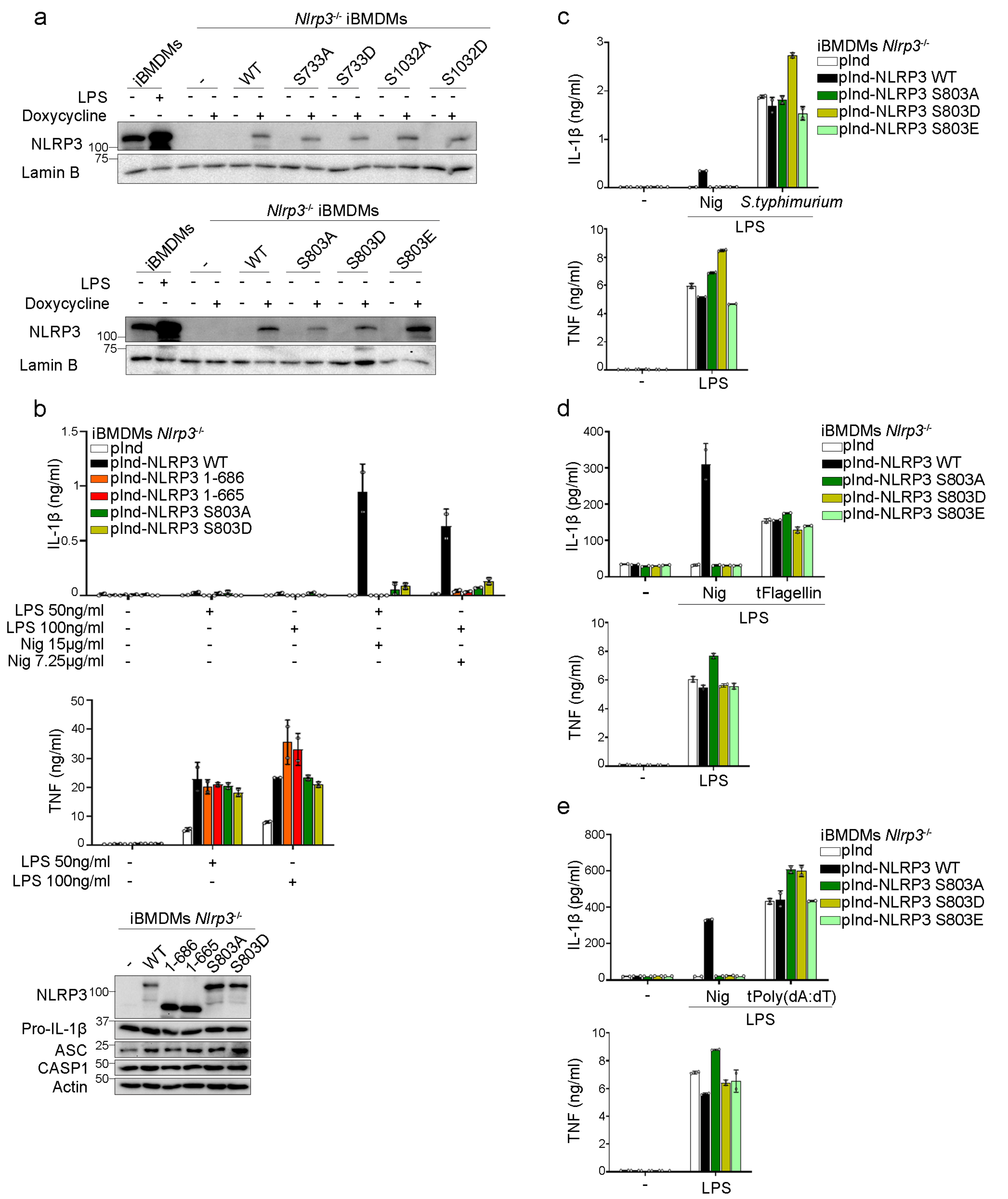

Supplementary Fig. 3. S803 is critical for NLRP3 inflammasome activity in immortalized BMDMs. **a** Immortalized WT and *Nlrp3*$^{-/-}$ BMDMs reconstituted with doxycycline-inducible NLRP3 mutants were treated with LPS (50 ng/ml, 4h) or doxycycline (2 μg/ml, 4h) as indicated. NLRP3 expression was analyzed by WB. **b** Immortalized *Nlrp3*$^{-/-}$ BMDMs reconstituted with doxycycline-inducible NLRP3 mutants were treated with either doxycycline (2 μg/ml, 16h) and then with LPS (50 ng/ml, 4h) followed by nigericin (15 μg/ml, 1h), or with doxycycline (2 μg/ml) and LPS (100 ng/ml) for 11h, before media change and addition of nigericin (7.25 μg/ml, 1h). IL-1β and TNF secretions were measured by ELISA. NLRP3 expression was analyzed by WB. **c** Immortalized *Nlrp3*$^{-/-}$ BMDMs reconstituted with doxycycline-inducible NLRP3 mutants were treated with doxycycline (2 μg/ml, 16h), LPS (50 ng/ml, 4h) and infected with *S. Typhimurium* (MOI 234, 2h). IL-1β and TNF secretions were measured by ELISA. **d** Immortalized *Nlrp3*$^{-/-}$ BMDMs reconstituted with doxycycline-inducible NLRP3 mutants were treated with doxycycline (2 μg/ml, 16h), LPS (50 ng/ml, 4h) and transfected with flagellin (10 μg/ml, 6h). IL-1β and TNF secretions were measured by ELISA. **e** Immortalized *Nlrp3*$^{-/-}$ BMDMs reconstituted with doxycycline-inducible NLRP3 mutants were treated with doxycycline (2 μg/ml, 16h), LPS (50 ng/ml, 4h) and transfected with poly(dA:dT) (1 μg/ml, 6h) as indicated. IL-1β and TNF secretions were measured by ELISA. Means and 1 SD are shown. Data are representative of 2 independent experiments (a, b) and biological duplicates representative of 2 (c, d, e) and 3 (b) independent experiments. Molecular weights are indicated in kDa (a, b). iBMDMs, immortalized BMDMs; pInd, pInducer21; tPoly(dA:dT), transfected poly(dA:dT).

Supplementary Figure 4

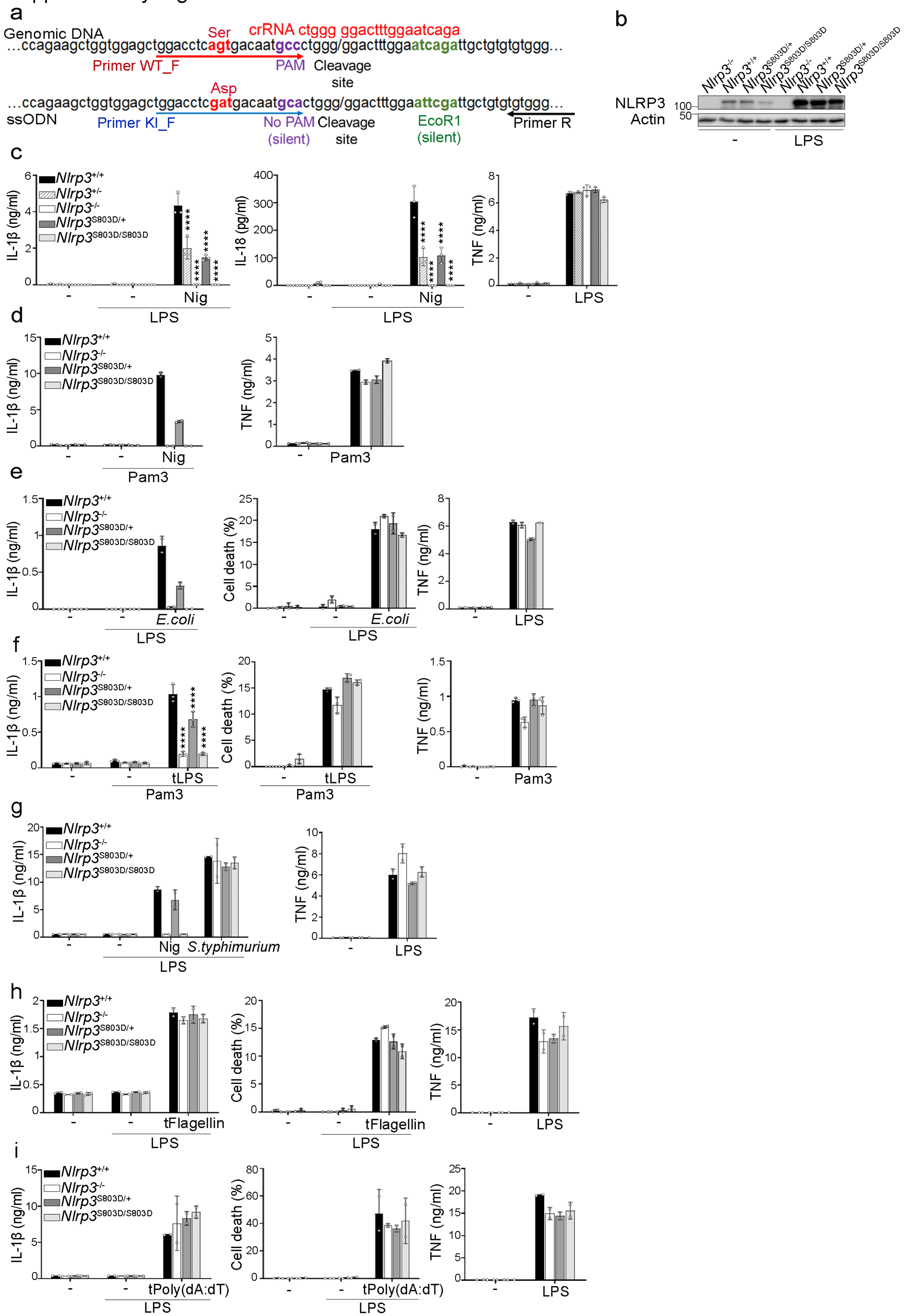

Supplementary Fig. 4. S803 is critical for NLRP3 inflammasome activity in primary BMDMs. **a** S803D mutagenesis strategy. CrRNA was designed to target Crispr/CAS9 cleavage in proximity to exon coding for S803. ssODN comprises 3 mutations: agT to gaT encoding the substitution of S803 to D (red), GCc to GCa silent mutation to remove the PAM sequence and prevent secondary cleavage (purple), ATcaGA to ATtcGA silent mutation to insert an EcoR1 site used for genotyping. Mice were genotyped by PCR using WT-F, KI-F specific primers with the R primer and confirmation by EcoR1 digestion pattern. **b** BMDMs from *Nlrp3*$^{S803D/S803D}$, *Nlrp3*$^{S803D/+}$ and *Nlrp3*$^{+/+}$ littermate mice were treated with LPS (50 ng/ml, 4h) and NLRP3 expression in cell lysates was assessed by WB. **c** BMDMs from *Nlrp3*$^{+/+}$, *Nlrp3*$^{+/-}$, *Nlrp3*$^{-/-}$, *Nlrp3*$^{S803D/+}$ and *Nlrp3*$^{S803D/S803D}$ mice were treated with LPS (50 ng/ml, 4h) followed by nigericin (15 μg/ml, 1h). **d** BMDMs from *Nlrp3*$^{S803D/S803D}$, *Nlrp3*$^{S803D/+}$ and *Nlrp3*$^{+/+}$ littermates were primed with Pam3CSK4 (1 μg/ml, 1h) followed by nigericin (15 μg/ml, 1h) treatment as indicated. **e** BMDMs from *Nlrp3*$^{S803D/S803D}$, *Nlrp3*$^{S803D/+}$ and *Nlrp3*$^{+/+}$ littermate mice were primed with LPS (50 ng/ml, 4h) followed by infection with *E. coli* (MOI 100, 16h). **f** BMDMs were primed with Pam3CSK4 (1 μg/ml, 4h) and then transfected with LPS (500 ng/ml, 20h). **g** BMDMs from *Nlrp3*$^{S803D/S803D}$, *Nlrp3*$^{S803D/+}$ and *Nlrp3*$^{+/+}$ littermate mice were primed with LPS (50 ng/ml, 4h) followed by infection with *S. Typhimurium* (MOI 234, 2h) or treatment with nigericin (15 μg/ml, 1h) as a control. **h** BMDMs from *Nlrp3*$^{S803D/S803D}$, *Nlrp3*$^{S803D/+}$ and *Nlrp3*$^{+/+}$ littermate mice were primed with LPS (50 ng/ml, 4h) followed by transfection with flagellin (10 μg/ml, 6h). **i** BMDMs from *Nlrp3*$^{S803D/S803D}$, *Nlrp3*$^{S803D/+}$ and *Nlrp3*$^{+/+}$ littermate mice were primed with LPS (50 ng/ml, 4h) followed by transfection with poly(dA:dT) (1 μg/ml, 6h). IL-1β and TNF

secretions in the supernatant were measured by ELISA. Cell death was monitored by LDH release. BMDMs from *Nlrp3*$^{-/-}$ mice were used as controls. Means and 1 SD are represented. Data are representative of 2 independent experiments (b) and biological duplicates representative of 2 (c-i) independent experiments. Molecular weights are indicated in kDa b). tLPS, transfected LPS; tFlagellin, transfected flagellin; tPoly(dA:dT), transfected poly(dA:dT).

Supplementary Figure 5

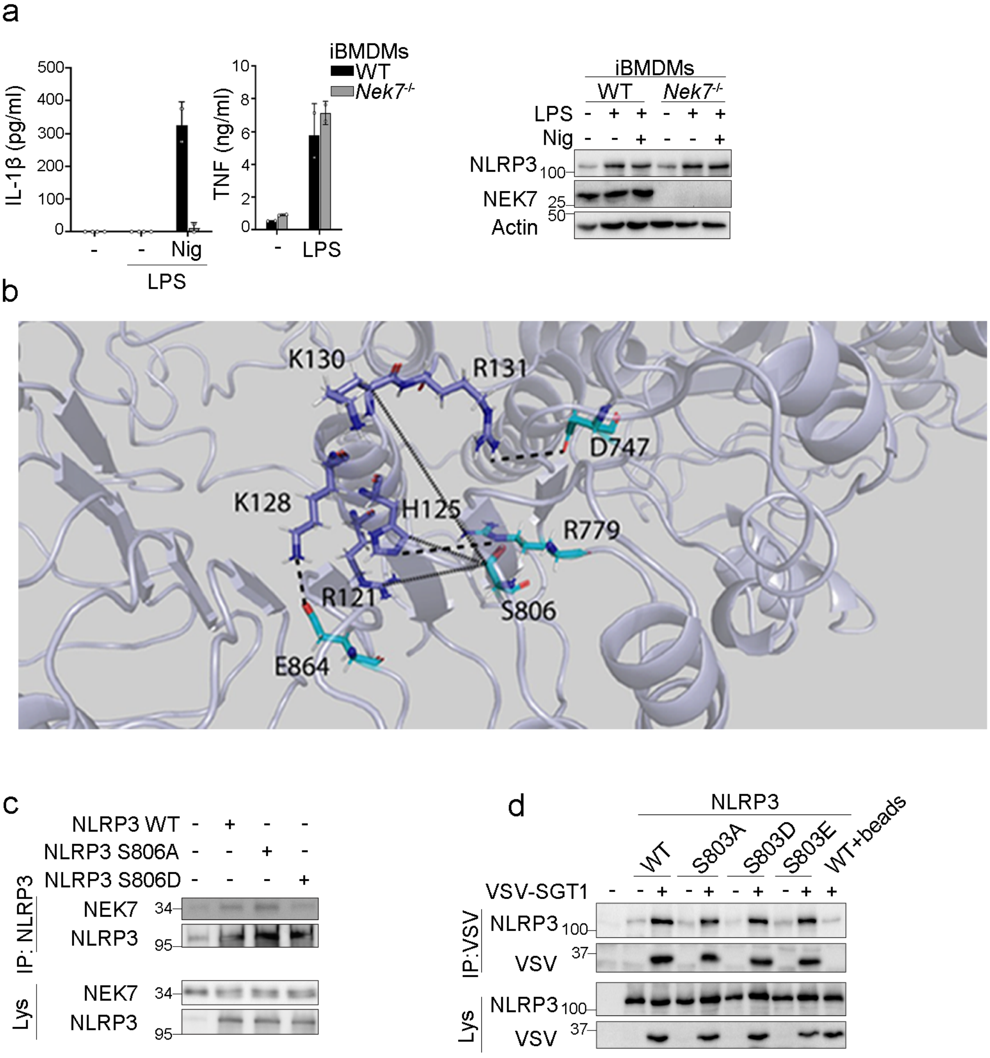

Supplementary Fig. 5. S803D substitution impairs NEK7 recruitment. **a** Immortalized WT and *Nek7*$^{-/-}$ BMDMs were treated with LPS (50 ng/ml, 4h) followed by nigericin (15 μg/ml, 1h). IL-1β and TNF secretions in the supernatant were measured by ELISA. NLRP3 and NEK7 protein level was analyzed by WB. Data are means and 1 SD of biological duplicates representative of 3 independent experiments. **b** Cartoon representation of the NLRP3/NEK7 complex around S806 using PyMOL software. Residues R121, H125, K128, K130 and R131 of NEK7 as well as D747, R779, S806 and E864 of NLRP3 are depicted in sticks. Distances between S806 and R131, H125 or K130 are depicted with short dotted lines and hydrogen bonds between D747-R131, R777-H125, E864-K128 are depicted with large dotted lines. **c** NLRP3 immunoprecipitates from lysates of HeLa cells expressing NLRP3 WT, S806D or S806A were analyzed for NEK7 by WB. **d** VSV-SGT1 immunoprecipitates from lysates of 293T cells expressing VSV-SGT1 and NLRP3 WT, S803A, S803D or S803E mutants were analyzed for NLRP3 by WB. Lysate of 293T expressing VSV-SGT1 and NLRP3 WT incubated with A/G-beads (WT+beads) was used as a negative control. Data are representative of 2 (d) and 3 (a, c) independent experiments. Molecular weights are indicated in kDa (a, c, d). iBMDMs, immortalized BMDMs; Lys, lysates; IP, immunoprecipitates.

Supplementary Figure 6

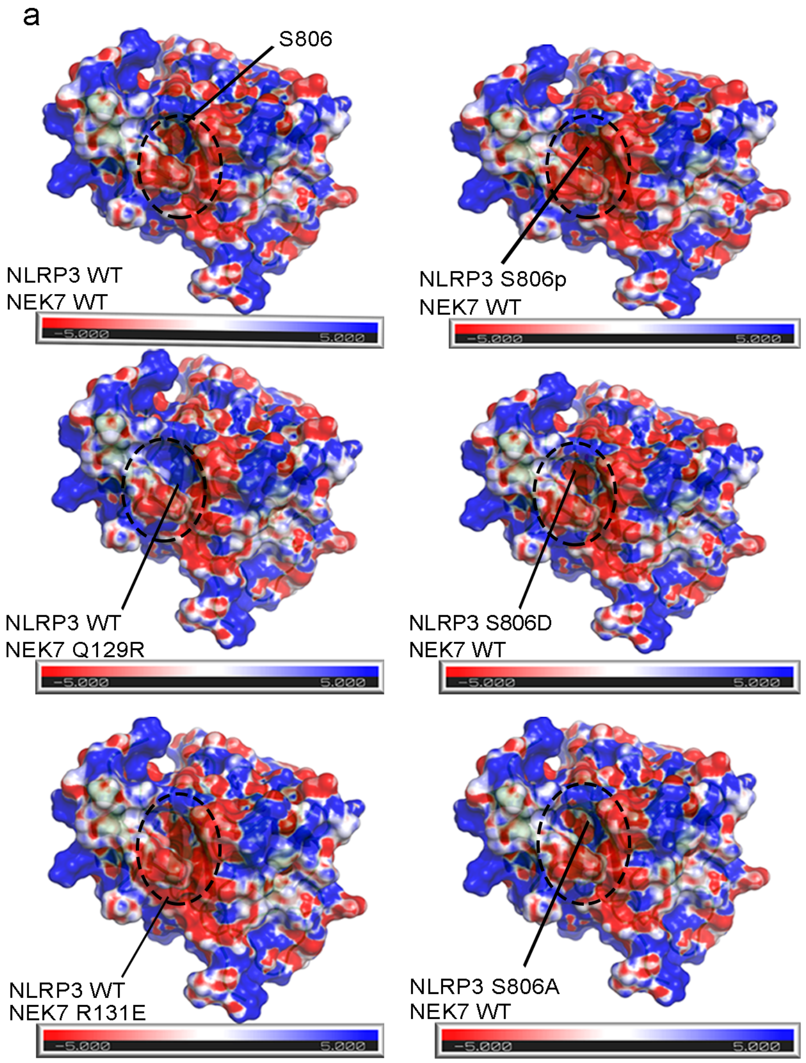

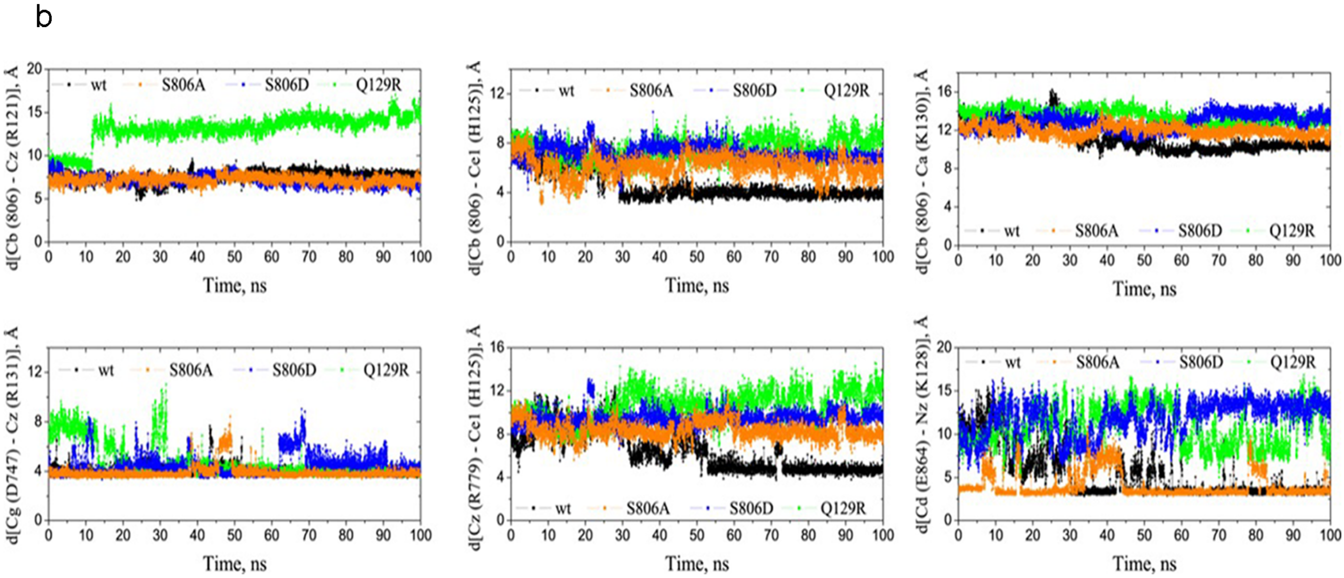

Supplementary Fig. 6. S803D substitution modifies NLRP3 interaction surface with NEK7. **a** Electrostatic potential surfaces of the NLRP3/NEK7 complexes with phosphorylated S806 (S806p), NLRP3 S806D, S806A and NEK7 Q129R, R131E. An ellipse is focused on the vicinity of the S806 of NLRP3. The position of each mutated residue is pointed on each structure. **b** Distances measurement (C_b (806)/C_z (R121), C_b (806)/C_{e1} (H125), C_b (806)/C_a (K130), C_g (D747)/C_z (R131), C_z (R779)/C_{e1} (H125) and C_d (E864)/N_z (K128)) along the 100 ns molecular dynamic trajectory for NLRP3/NEK7 complexes, and complexes with NLRP3 S806A, NLRP3 S806D and NEK7 Q129R.

Supplementary Figure 7

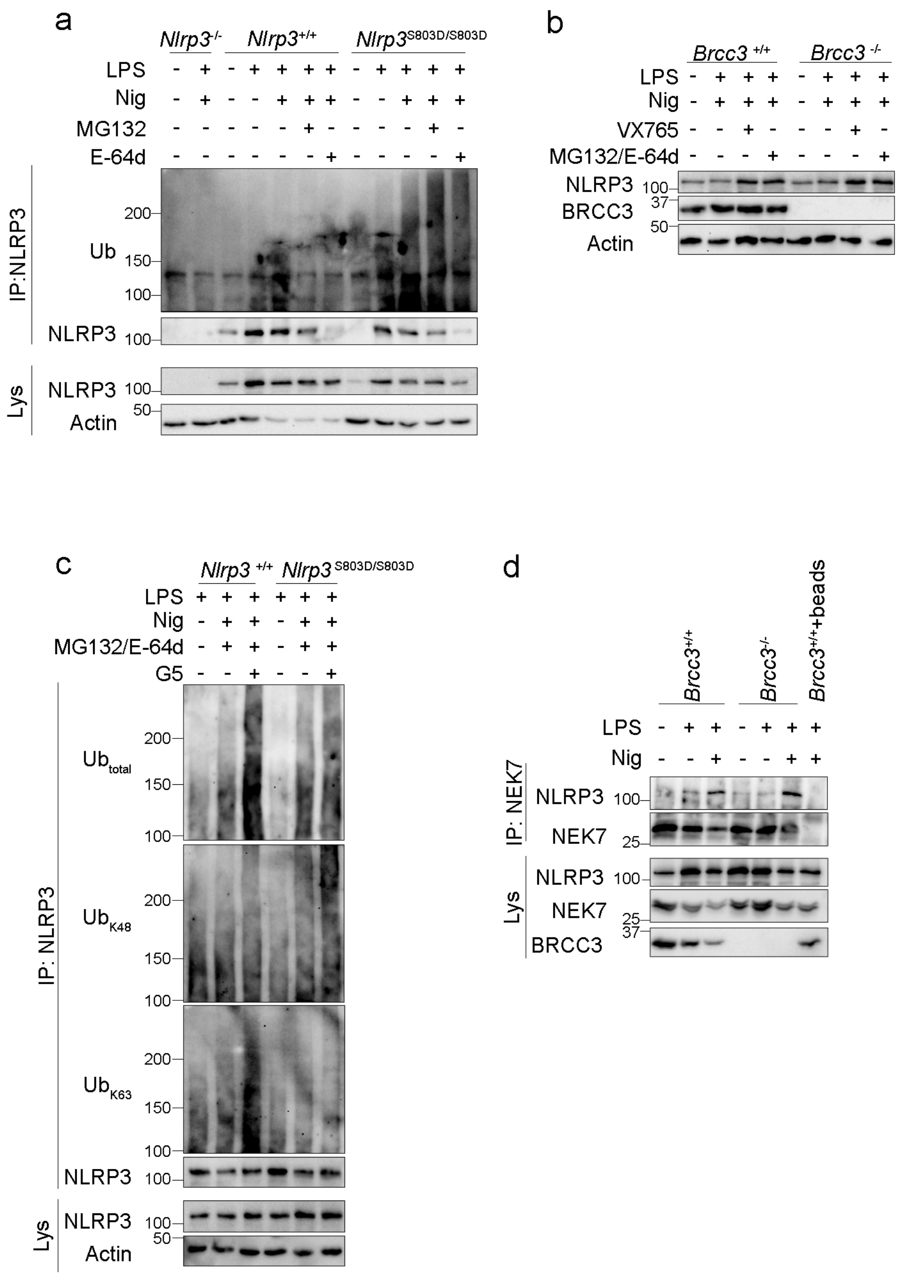

Supplementary Fig. 7. S803D substitution leads to degradative K48 ubiquitination following activation signal. **a** BMDMs from *Nlrp3*$^{S803D/S803D}$, *Nlrp3*$^{+/+}$ and *Nlrp3*$^{-/-}$ mice were primed with LPS (50 ng/ml, 6h) and then treated with nigericin (15 μg/ml, 45 min) 15 min before the addition of MG132 (10 μM, 30 min) and E-64d (20 μg/ml, 30 min). NLRP3 ubiquitination was assessed by NLRP3 immunoprecipitation followed by anti-Ub WB. **b** BMDMs from *Brcc3*$^{-/-}$ and *Brcc3*$^{+/+}$ littermate mice were primed with LPS (50 ng/ml, 5h) followed by nigericin (15 μg/ml, 1h) treatment. Cells were treated with VX765 (2.5 μM), MG132 (10 μM) and/or E-64d (20 μg/ml) 15 min before nigericin. NLRP3 and BRCC3 protein levels were analyzed by WB. **c** BMDMs from WT and *Nlrp3*$^{S803D/S803D}$ mice were primed with LPS (50 ng/ml, 5h) followed by nigericin (15 μg/ml, 1h) treatment. Cells were treated with MG132 (10 μM), E-64d (20 μg/ml) or G5 (1 μM) 15 min before nigericin. NLRP3 immunoprecipitates were analyzed for K63 Ub, K48 Ub and total Ub by WB. **d** BMDMs from *Brcc3*$^{-/-}$, and *Brcc3*$^{+/+}$ littermate mice were primed with LPS (50 ng/ml, 6h) followed by nigericin (15 μg/ml, 30 min) treatment. Endogenous NEK7 immunoprecipitates were analyzed for NLRP3 by WB. Lysate of *Brcc3*$^{+/+}$ BMDMs incubated with A/G-beads (*Brcc3*$^{+/+}$+beads) was used as a negative control. Data are representative of 2 (a, b, d) and 3 (c) independent experiments. Molecular weights are indicated in kDa (a-d). Lys, lysates; IP, immunoprecipitates; Ub, ubiquitin.

Supplementary Figure 8

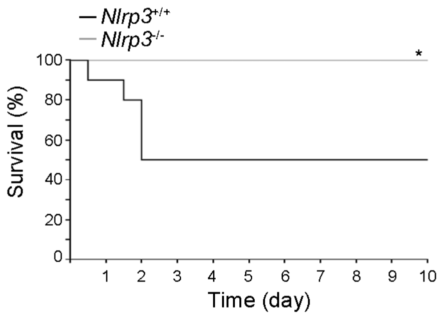

Supplementary Fig. 8. *Nlrp3*$^{-/-}$ mice show an impaired response to endotoxic shock in vivo. *Nlrp3*$^{-/-}$ (n=7) and *Nlrp3*$^{+/+}$ (n=10) littermates were injected intraperitoneally with LPS (10 mg/kg). Two-sided Mantel-Cox test, *, $p<0.05$. Data are biological replicates of one experiment.

Supplementary Figure 9

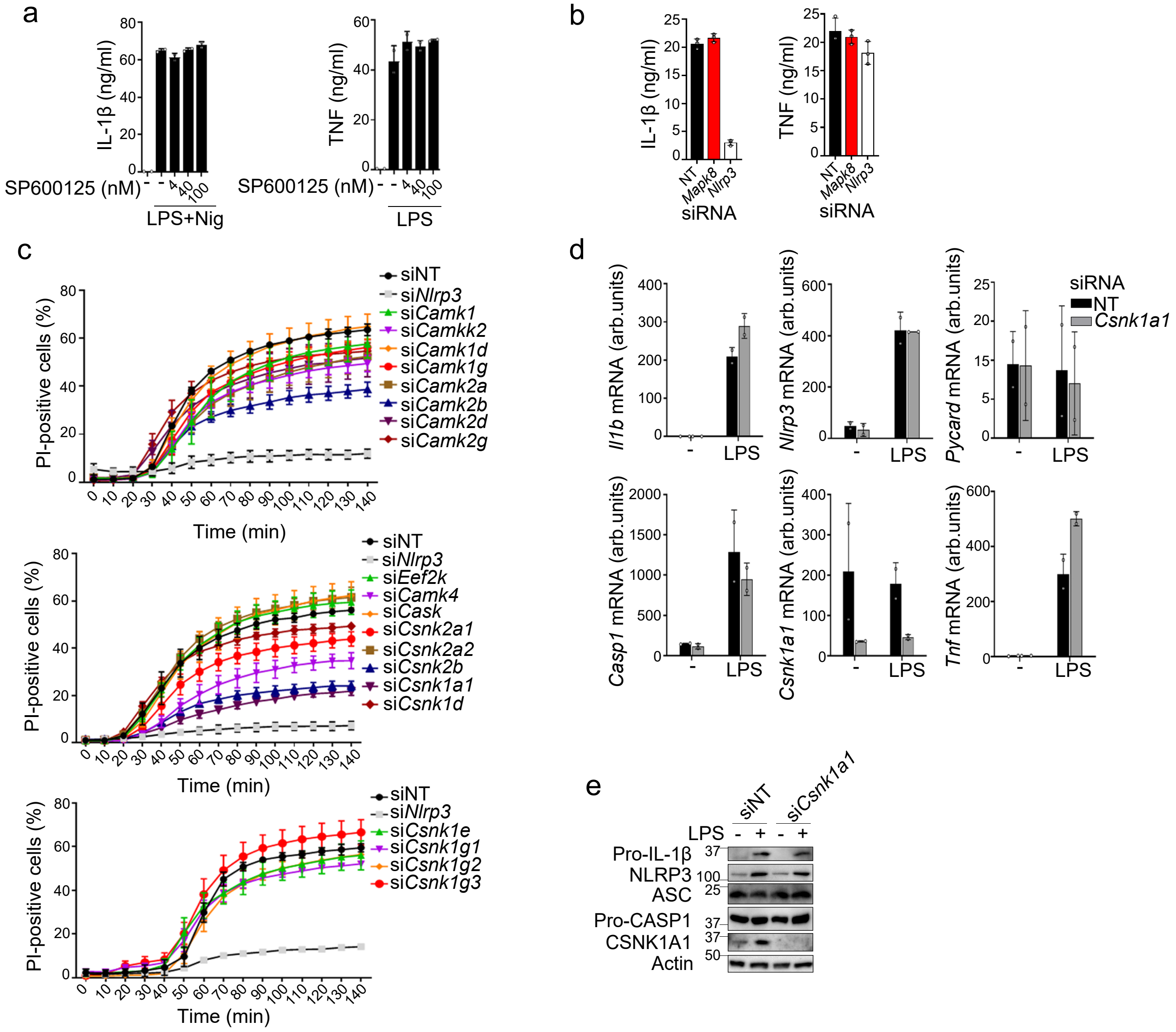

Supplementary Fig. 9. CSNK1A1 phosphorylates NLRP3 at S806. **a** BMDMs were treated with SP600125 15 min prior priming with LPS (50 ng/ml, 4h) followed by treatment with nigericin (15 mg/ml, 1h). IL-1β and TNF secretions were measured by ELISA. **b** BMDMs transfected with the indicated siRNAs were treated with LPS (50 ng/ml, 4h) followed by nigericin (15 μg/ml, 1h). IL-1β and TNF secretions were measured by ELISA. **c** BMDMs were transfected with the indicated siRNAs and treated with LPS (50 ng/ml, 4h) followed by nigericin (15 μg/ml). Cell death was monitored by PI incorporation over time quantified by high content microscopy. **d** BMDMs were transfected with siRNA targeting *Csnk1a1* and treated with LPS (50 ng/ml, 6h). Relative gene expressions of each indicated gene were analyzed by qPCR using *Gapdh* and *Hprt* as reference genes. Means and 1 SD of two independent experiments done in duplicate are represented. Non-targeting siRNAs were used as controls. **e** Pro-IL-1β, NLRP3, ASC, Caspase-1, CSNK1A1 and TNF protein levels in cell lysate were analyzed by WB. Means and 1 SD are represented. Data are biological duplicates representative of 2 independent experiments (a, b), biological quadruplicates representative of 3 independent experiments (c), means of 2 independent experiments (d) or representative of 2 independent experiments (e). Molecular weights are indicated in kDa (e). NT, non-targeting siRNA; arb.units, arbitrary unit.

Supplementary Figure 10

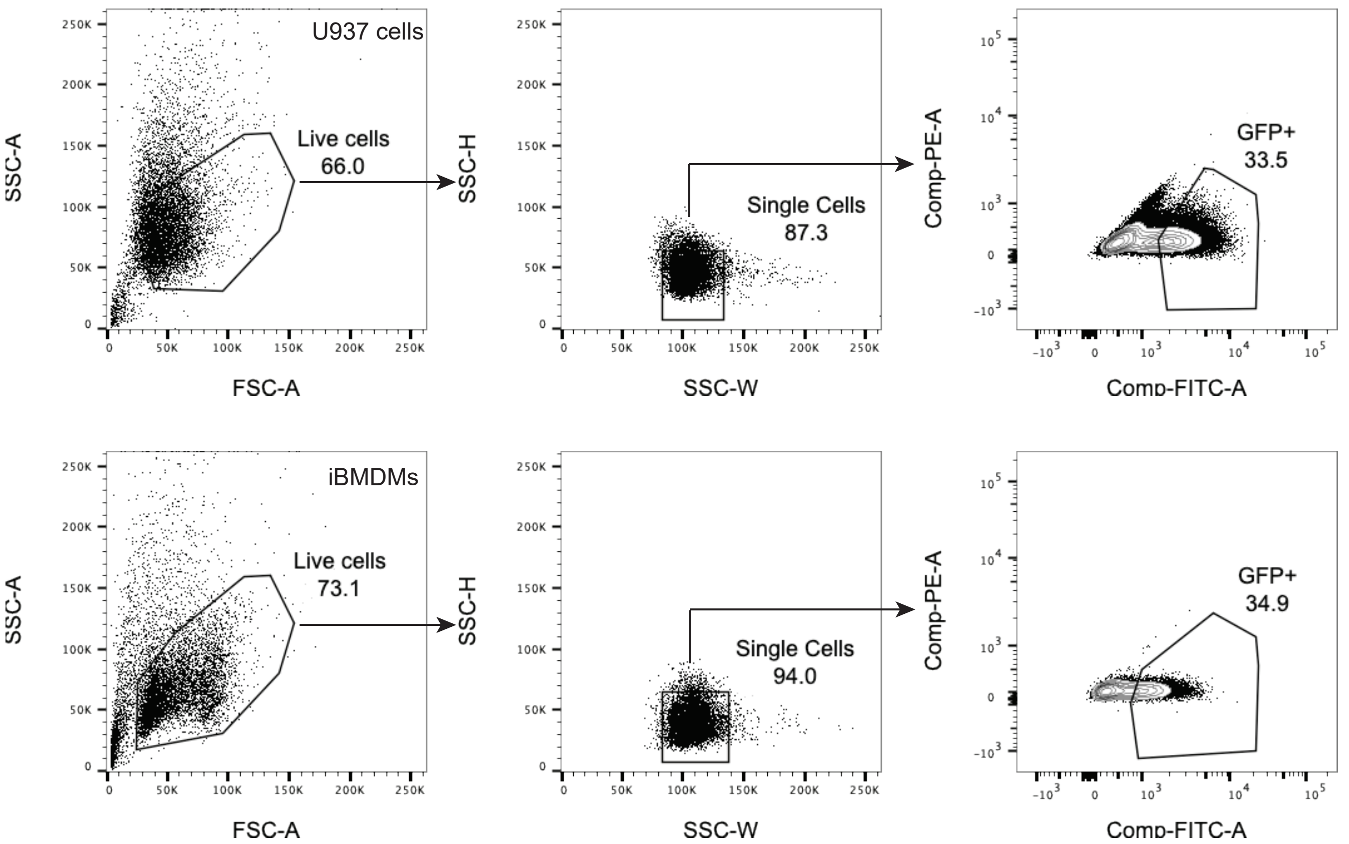

Supplementary Fig. 10. FACS sequential gating and GFP^+ cell sorting strategies to select NLRP3-deficient U937 cells (upper panel) and *Nlrp3*$^{-/-}$ immortalized BMDMs (lower panel) reconstituted with pInducer21-NLRP3 constructs. Percentages in the parent populations are indicated. iBMDMs, immortalized BMDMs.

	NLRP3/NEK7<br>15 H bounds	NLRP3 S806A/NEK7<br>20 H bounds	NLRP3 S806D/NEK7<br>9 H bounds	NLRP3/NEK7 Q129R<br>9 H bounds	NLRP3/NEK7 R131E<br>11 H bounds
LRR	D747-R131<br>E864-K128<br>R779-H125<br>D807-R121<br>Y1009-A193<br>Y1009-H194<br>W833-Q129<br>E1007-R184<br>S1035-T199<br>Q638-E265<br>D641-R294	D747-R131<br>E864-K128<br>R779-A116<br>R779-I169<br>D807-R121<br>K696-D261<br>E636-R268<br>Q638-E265<br>E640-E266<br>D641-C298<br>D641-R294<br>S1035-K127<br>K1015-M241<br>E1033-Y237<br>W1036-S234	D747-R131<br>E864-R121<br>R779-T170<br>E1007-K127<br>Y1012-M241<br>R920-H125<br>D867-R121	D747-R131<br>E864-R121<br>E1033-K240<br>D980-K189<br>D804-R129<br>D750-R136	D747-R136<br>E864-R121<br>D807-R121<br>R920-H125<br>E640-E265<br>V1030-Y237<br>E1033-M241<br>S1035-L243
NACHT	Q181-K140<br>Q181-T172<br>R183-A171<br>E356-R294	E214-H151<br>E214-R155<br>E217-R155<br>R178-A297<br>E176-K293	E214-R155<br>E356-R294	E356-R294<br>E182-T299<br>Q185-T299	S216-E150<br>E217-R155<br>E176-R294

Supplementary Table 1. Molecular dynamics of NLRP3-NEK7 complex. List of the hydrogen bonds for each structure after 100 ns of molecular dynamic simulations

Kinase family	Kinase subfamily	Official symbol		Gene ID	
		Mouse	Human	Mouse	Human
CAMK	CAMK1	Camk1	CAMK1	52163	8536
		Camkk2	CAMKK2	207565	10645
		Camk1d	CAMK1D	227541	57118
		Camk1g	CAMK1G	215303	57172
	CAMK2	Camk2a	CAMK2A	12322	815
		Camk2b	CAMK2B	12323	816
		Camk2d	CAMK2D	108058	817
		Camk2g	CAMK2G	12325	818
	CAMKIII	Eef2k	EEF2K	13631	29904
	CAMKIV	Camk4	CAMK4	12326	814
	CASK	Cask	CASK	12361	8573
CK2 (CSNK2)		Csnk2a1	CSNK2A1	12995	1457
		Csnk2a2	CSNK2A2	13000	1459
		Csnk2b	CSNK2B	13001	1460
CK1 (CSNK1)		Csnk1a1	CSNK1A1	93687	1452
		Csnk1d	CSNK1D	104318	1453
		Csnk1e	CSNK1E	27373	1454
		Csnk1g1	CSNK1G1	214897	53944
		Csnk1g2	CSNK1G2	103236	1455
		Csnk1g3	CSNK1G3	70425	1456

Supplementary Table 2. Identification of 20 kinase candidates with consensus targeting site matching NLRP3 S803. NLRP3 S806 phosphorylation site was analyzed by GPS 3.0 and NetPhos 2.0 softwares to identify putative kinases with compatible consensus targeting sites. Their indicated mouse orthologs were then screened using siRNA for their impact on NLRP3 activity.

Name	Sequence	Application
hNLRP3_S735A_F	5'-gccggggcctctttGcagttctgagcaccagcc-3'	cloning
hNLRP3_S735A_R	5'-ggctggtgctcagaactgCaaagaggccccggc-3'	cloning
hNLRP3_S735D_F	5'-gccggggcctctttGATgttctgagcaccagcc-3'	cloning
hNLRP3_S735D_R	5'-ggctggtgctcagaacATCaaagaggccccggc-3'	cloning
mNLRP3_S733A_F	5'-gccgtggtctcttcGcaagtctaagcaccaacc-3'	cloning
mNLRP3_S733A_R	5'-ggttggtgcttagacttgCgaagagaccacggc-3'	cloning
mNLRP3_S733D_F	5'-ctgccgtggtctcttcGATagtctaagcaccaaccgg-3'	cloning
mNLRP3_S733D_R	5'-ccggttggtgcttagactATCgaagagaccacggcag-3'	cloning
hNLRP3_S806A_F	5'-gctggtggagctggacctgGCtgacaacgccctcgg-3'	cloning
hNLRP3_S806A_R	5'-ccgagggcgttgtcaGCcaggtccagctccaccagc-3'	cloning
hNLRP3_S806D_F	5'-gctggtggagctggacctgGAtgacaacgccctcgg-3'	cloning
hNLRP3_S806D_R	5'-ccgagggcgttgtcaTCcaggtccagctccaccagc-3'	cloning
mNLRP3_S803A_F	5'-ggtggagctggacctcGCtgacaatgccctgg-3'	cloning
mNLRP3_S803A_R	5'-ccagggcattgtcaGCgaggtccagctccacc-3'	cloning
mNLRP3_S803D_F	5'-ggtggagctggacctcGAtgacaatgccctgg-3'	cloning
mNLRP3_S803D_R	5'-ccagggcattgtcaTCgaggtccagctccacc-3'	cloning
hNLRP3_S806E_F	5'-gtggagctggacctgGAGgacaacgccctcgg-3'	cloning
hNLRP3_S806E_R	5'-ccgagggcgttgtcCTCcaggtccagctccac-3'	cloning
mNLRP3_S803E_F	5'-gtggagctggacctcGAGgacaatgccctgggg-3'	cloning
mNLRP3_S803E_R	5'-ccccagggcattgtcCTCgaggtccagctccac-3'	cloning
mNLRP3_S1032A_F	5'-tatagtcttcgagattGcctggtagctcgagatatc-3'	cloning
mNLRP3_S1032A_R	5'-gatatctcgagctaccaggCaatctcgaagactata-3'	cloning
mNLRP3_S1032D_F	5'-gactatagtcttcgagattGActggtagctcgagatatctag-3'	cloning
mNLRP3_S1032D_R	5'-ctagatatctcgagctaccagTCaatctcgaagactatagtc-3'	cloning
hNLRP3_1F_BamHI	5'-CGTCGTCGAGGATCCACCACCATGaagatggcaagcacccgc-3'	cloning
hNLRP3_1995StopR_XhO1(1-667)	5'-TGCAAACGGctcgagctaGGAAGAAACCATGTGGTC-3'	cloning
hNLRP3_2058StopR_XhO1(1-688)	5'-TGCAAACGGctcgagctaGGGCATGTTATGGAGAAA-3'	cloning
mNLRP3_1F_Kpn1	5'-CGTCGTCGGGGTACCACCATGacgagtgtccgttgcaag-3'	cloning
mNLRP3_1995StopR_XhO1(1-665)	5'-TGCAAACGGctcgagctaGGAGGAAACCACGTGGTC-3'	cloning
mNLRP3_2058StopR_XhO1(1-686)	5'-TGCAAACGGctcgagctaGGGCGAGTTGTGAAAAAA-3'	cloning
mHprt_F	5'-GGGCTTACCTCACTGCTTTCC-3'	qPCR
mHprt_R	5'-TCATCGCTAATCACGACGCTG-3'	qPCR
mGapdh_F	5'-GGGTTCCTATAAATACGGACTGC-3'	qPCR
mGapdh_R	5'-CTGGCACTGCACAAGAAGAT-3'	qPCR
mIl-1β_F	5'-TAACCTGCTGGTGTGTGACG-3'	qPCR
mIl-1β_R	5'-GCTTGTGCTCTGCTTGTGAG-3'	qPCR
mTnf_F	5'-ACGCTCTTCTGTCTACTGAACT-3'	qPCR
mTnf_R	5'-ATCTGAGTGTGAGGGTCTGG-3'	qPCR
mNlrp3_F	5'-GATTACCCGCCCGAGAAAGG-3'	qPCR
mNlrp3_R	5'-CTTCTCCTCGCCATTGAAGT-3'	qPCR
mPycard_F	5'-TTAATCCCAGCAACCAGGAG-3'	qPCR
mPycard_R	5'-CTTGAGTTAGGCCAGCCTTG-3'	qPCR
mCasp1_F	5'-CACAGCTCTGGAGATGGTGA-3'	qPCR
mCasp1_R	5'-CTTTCAAGCTTGGGCACTTC-3'	qPCR
mCsnk1a1_F	5'-AAGGCCGAATTTATCGTCGGT-3'	qPCR
mCsnk1a1_R	5'-ACTTCCTCGCCATTGGTGATG-3'	qPCR

Supplementary Table 3. Primers.

Antibody	Supplier	Cat Number	Clone	Lot	Dilution
anti-Actin	Sigma-Aldrich	MAB1501	C4	3590048	1/10000 (WB)
anti-ASC	Adipogen	AG-25B-0006-C100	AL177	A40922001	1/1000 (WB, IF), 1/300 (IP)
anti-ASC (N-15)	Santa Cruz Biotechnology	sc-22514-R		I0712	1/1000 (WB, IF),
anti-BRCC3	Cell Signaling Technology	18215S	D5E5H	1	1/1000 (WB), 1/300 (IP)
anti-Caspase-1	BioLegend	645102	5B10	B257128	1/333 (WB)
anti-CK1 alpha	Bethyl Laboratories	A301-991A		1	1/500 (WB), 1/300 (IP)
anti-CK1 alpha	Santa Cruz Biotechnology	sc-74582	H-7	J0620	1/100 (WB)
anti-Flag	Sigma-Aldrich	F3165	M2	SLCG2330	1/500 (WB)
anti-HA	Sigma-Aldrich	H3663	HA-7	092M4827V	1/1000 (WB)
anti-IL-1b	R&D Systems	MAB4011	166926		1/500 (WB)
anti-IL-1b	Cell Signaling Technology	12703	D3U3E	2	1/1000 (WB)
anti-LaminB1 (B-10)	Santa Cruz Biotechnology	sc-374015		F1014	1/200 (WB)
anti-Myc	Sigma-Aldrich	C3956			1/500 (WB), 1/300 (IP)
anti-NEK7	Abcam	ab133514	EPR4900	GR3258619-1	1/1000 (WB), 1/300 (IP)
anti-NLRP3	Adipogen	AG-20B-0014-C100	Cryo2	A41812012	1/500 (WB), 1/400 (IP)
anti-pNLRP3$_{S198}$	From Dr. Tao Li, Beijing China	N/A			1/1000 (WB)
anti-Ubiquitin	Dako	Z0458		94251	1/200 (WB)
anti-Ubiquitin	Santa Cruz Biotechnology	sc-8017		H2719	1/1000 (WB)
anti-Ubiquitin	Cell Signaling Technology	3933		6	1/1000 (WB)
anti-Ubiquitin (K48)	Cell Signaling Technology	4289		2	1/1000 (WB)
anti-Ubiquitin (K63)	Cell Signaling Technology	5621		5	1/1000 (WB)
anti-VSV	Sigma-Aldrich	V5507	P5D4	035M4777U	1/5000 (WB), 1/300 (IP)
anti-phospho(Ser/Thr)	Cell Signaling Technology	9631S		10	1/1000 (WB)
anti-Mouse IgG (H+ L)-HRP	Promega	W402B		00441155	1/10000 (WB)
anti-Rabbit IgG (H+L)-HRP	Promega	W401B		390794	1/10000 (WB)
anti-Rat IgM+IgG (H+L)-HRP	Southern Biotech	3010-05		G2512-M748B	1/3000 (WB)
anti-Rabbit IgG (H+L)-HRP	Invitrogen	A21074			1/3000 (WB)
IgG	Diagenode	C15410206		RIG001AM	1/300 (IP)
anti-Flag agarose	Sigma-Aldrich	A22220	M2		1/30 (IP)
anti-HA agarose	Sigma-Aldrich	A2095	HA-7	048M4893V	1/30 (IP)

Supplementary Table 4. Antibodies.

	Treatment	Conditons	Adjusted P Values	Significance
Fig. 2d	Ordinary two-way ANOVA with Tukey's multiple comparisons			
	LPS	pInd-NLRP3 WT vs. pInd	0.9907	ns
		pInd-NLRP3 WT vs. pInd-NLRP3 S806D	0.9915	ns
	LPS+Nig	pInd-NLRP3 WT vs. pInd	<0.0001	****
		pInd-NLRP3 WT vs. pInd-NLRP3 S806D	<0.0001	****
Fig. 4b	Repeated measure two-way ANOVA with Sidak's multiple comparisons			
	0 min	*Nlrp3*$^{+/+}$ vs. *Nlrp3*$^{S803D/+}$	0.0971	ns
		Nlrp3$^{+/+}$ vs. *Nlrp3*$^{S803D/S803D}$	0.1296	ns
		Nlrp3$^{+/+}$ vs. *Nlrp3*$^{-/-}$	0.0665	ns
	10 min	*Nlrp3*$^{+/+}$ vs. *Nlrp3*$^{S803D/+}$	0.0708	ns
		Nlrp3$^{+/+}$ vs. *Nlrp3*$^{S803D/S803D}$	0.0534	ns
		Nlrp3$^{+/+}$ vs. *Nlrp3*$^{-/-}$	0.0461	*
	20 min	*Nlrp3*$^{+/+}$ vs. *Nlrp3*$^{S803D/+}$	0.0337	*
		Nlrp3$^{+/+}$ vs. *Nlrp3*$^{S803D/S803D}$	0.0201	*
		Nlrp3$^{+/+}$ vs. *Nlrp3*$^{-/-}$	0.0198	*
	30 min	*Nlrp3*$^{+/+}$ vs. *Nlrp3*$^{S803D/+}$	0.0012	**
		Nlrp3$^{+/+}$ vs. *Nlrp3*$^{S803D/S803D}$	0.0015	**
		Nlrp3$^{+/+}$ vs. *Nlrp3*$^{-/-}$	0.0015	**
	40 min	*Nlrp3*$^{+/+}$ vs. *Nlrp3*$^{S803D/+}$	0.0005	***
		Nlrp3$^{+/+}$ vs. *Nlrp3*$^{S803D/S803D}$	0.0005	***
		Nlrp3$^{+/+}$ vs. *Nlrp3*$^{-/-}$	0.0005	***
	50 min	*Nlrp3*$^{+/+}$ vs. *Nlrp3*$^{S803D/+}$	0.0004	***
		Nlrp3$^{+/+}$ vs. *Nlrp3*$^{S803D/S803D}$	0.0002	***
		Nlrp3$^{+/+}$ vs. *Nlrp3*$^{-/-}$	0.0002	***
	60 min	*Nlrp3*$^{+/+}$ vs. *Nlrp3*$^{S803D/+}$	0.0007	***
		Nlrp3$^{+/+}$ vs. *Nlrp3*$^{S803D/S803D}$	0.0001	***
		Nlrp3$^{+/+}$ vs. *Nlrp3*$^{-/-}$	0.0001	***
	70 min	*Nlrp3*$^{+/+}$ vs. *Nlrp3*$^{S803D/+}$	0.0010	***
		Nlrp3$^{+/+}$ vs. *Nlrp3*$^{S803D/S803D}$	<0.0001	****
		Nlrp3$^{+/+}$ vs. *Nlrp3*$^{-/-}$	<0.0001	****
	80 min	*Nlrp3*$^{+/+}$ vs. *Nlrp3*$^{S803D/+}$	0.0010	**
		Nlrp3$^{+/+}$ vs. *Nlrp3*$^{S803D/S803D}$	<0.0001	****
		Nlrp3$^{+/+}$ vs. *Nlrp3*$^{-/-}$	<0.0001	****
	90 min	*Nlrp3*$^{+/+}$ vs. *Nlrp3*$^{S803D/+}$	0.0007	***
		Nlrp3$^{+/+}$ vs. *Nlrp3*$^{S803D/S803D}$	<0.0001	****
		Nlrp3$^{+/+}$ vs. *Nlrp3*$^{-/-}$	<0.0001	****
	100 min	*Nlrp3*$^{+/+}$ vs. *Nlrp3*$^{S803D/+}$	0.0028	**
		Nlrp3$^{+/+}$ vs. *Nlrp3*$^{S803D/S803D}$	0.0001	***
		Nlrp3$^{+/+}$ vs. *Nlrp3*$^{-/-}$	<0.0001	****
	110 min	*Nlrp3*$^{+/+}$ vs. *Nlrp3*$^{S803D/+}$	0.0043	**
		Nlrp3$^{+/+}$ vs. *Nlrp3*$^{S803D/S803D}$	<0.0001	****
		Nlrp3$^{+/+}$ vs. *Nlrp3*$^{-/-}$	<0.0001	****
Fig. 4d	Ordinary two-way ANOVA with Tukey's multiple comparisons			
	LPS+Nig	*Nlrp3*$^{+/+}$ vs. *Nlrp3*$^{-/-}$	<0.0001	****
		Nlrp3$^{+/+}$ vs. *Nlrp3*$^{S803D/S803D}$	<0.0001	****
Fig. 4f	Ordinary two-way ANOVA with Tukey's multiple comparisons			
	-	*Nlrp3*$^{+/+}$ vs. *Nlrp3*$^{-/-}$	>0.9999	ns
		Nlrp3$^{+/+}$ vs. *Nlrp3*$^{S803D/S803D}$	>0.9999	ns
	LPS	*Nlrp3*$^{+/+}$ vs. *Nlrp3*$^{-/-}$	>0.9999	ns
		Nlrp3$^{+/+}$ vs. *Nlrp3*$^{S803D/S803D}$	>0.9999	ns
	LPS+Nig	*Nlrp3*$^{+/+}$ vs. *Nlrp3*$^{-/-}$	<0.0001	****
		Nlrp3$^{+/+}$ vs. *Nlrp3*$^{S803D/S803D}$	<0.0001	****
Fig. 6a	Repeated measure two-way ANOVA with Sidak's multiple comparisons			

IL-1b	0 h	*Nlrp3*$^{+/+}$ vs. *Nlrp3*$^{S803D/S803D}$	>0.9999	ns
	2 h	*Nlrp3*$^{+/+}$ vs. *Nlrp3*$^{S803D/S803D}$	0.0318	*
	4h	*Nlrp3*$^{+/+}$ vs. *Nlrp3*$^{S803D/S803D}$	<0.0001	****
IL-18	0 h	*Nlrp3*$^{+/+}$ vs. *Nlrp3*$^{S803D/S803D}$	0.7305	ns
	2 h	*Nlrp3*$^{+/+}$ vs. *Nlrp3*$^{S803D/S803D}$	0.0428	*
	4h	*Nlrp3*$^{+/+}$ vs. *Nlrp3*$^{S803D/S803D}$	<0.0001	****
IL-1a	0 h	*Nlrp3*$^{+/+}$ vs. *Nlrp3*$^{S803D/S803D}$	0.9675	ns
	2 h	*Nlrp3*$^{+/+}$ vs. *Nlrp3*$^{S803D/S803D}$	0.0506	ns
	4h	*Nlrp3*$^{+/+}$ vs. *Nlrp3*$^{S803D/S803D}$	0.0170	*
TNF	0 h	*Nlrp3*$^{+/+}$ vs. *Nlrp3*$^{S803D/S803D}$	>0.9999	ns
	2 h	*Nlrp3*$^{+/+}$ vs. *Nlrp3*$^{S803D/S803D}$	0.2815	ns
	4h	*Nlrp3*$^{+/+}$ vs. *Nlrp3*$^{S803D/S803D}$	>0.9999	ns
IL-6	0 h	*Nlrp3*$^{+/+}$ vs. *Nlrp3*$^{S803D/S803D}$	>0.9999	ns
	2 h	*Nlrp3*$^{+/+}$ vs. *Nlrp3*$^{S803D/S803D}$	0.9687	ns
	4h	*Nlrp3*$^{+/+}$ vs. *Nlrp3*$^{S803D/S803D}$	0.8174	ns
Fig. 6b	Two-sided Mantel-Cox test			
	LPS	*Nlrp3*$^{+/+}$ vs. *Nlrp3*$^{S803D/S803D}$	0.0030	**
Fig. 7a	Ordinary one-way ANOVA with Dunnett's multiple comparison test with single pooled variance			
	LPS+Nig	NT vs. *Csnk1g3*	0.4756	ns
		NT vs. *Csnk2a2*	0.7862	ns
		NT vs. *Csnk1g2*	0.9878	ns
		NT vs. *Cask*	0.9947	ns
		NT vs. *Eef2k*	0.9959	ns
		NT vs. *Camk1d*	0.9996	ns
		NT vs. *Csnk1d*	0.9727	ns
		NT vs. *Camk2g*	0.9161	ns
		NT vs. *Csnk1e*	0.7138	ns
		NT vs. *Camk1g*	0.1375	ns
		NT vs. *Camk1*	0.1280	ns
		NT vs. *Camk2d*	0.1001	ns
		NT vs. *Csnk1g1*	0.0103	*
		NT vs. *Camk2a*	0.0048	**
		NT vs. *Csnk2a1*	0.0034	**
		NT vs. *Camkk2*	0.0016	**
		NT vs. *Camk2b*	0.0001	****
		NT vs. *Camk4*	0.0001	****
		NT vs. *Csnk2b*	0.0001	****
		NT vs. *Csnk1a1*	0.0001	****
		NT vs. *Nlrp3*	0.0001	****
Fig. 7c	Ordinary two-way ANOVA with Tukey's multiple comparisons			
IL-18	-	NT vs. *Csnk1a1*	0.9902	ns
		NT vs. *Nlrp3*	0.9660	ns
	LPS	NT vs. *Csnk1a1*	0.9703	ns
		NT vs. *Nlrp3*	0.7459	ns
	LPS+Nig	NT vs. *Csnk1a1*	<0.0001	****
		NT vs. *Nlrp3*	<0.0001	****
TNF	-	NT vs. *Csnk1a1*	0.9827	ns
		NT vs. *Nlrp3*	0.9836	ns
	LPS	NT vs. *Csnk1a1*	0.0841	ns
		NT vs. *Nlrp3*	0.8889	ns
Fig. 7d	Ordinary two-way ANOVA with Tukey's multiple comparisons			
	LPS	NT vs. *Csnk1a1*	>0.9999	ns
	LPS+Nig	NT vs. *Csnk1a1*	<0.0001	****
Sup Fig. 4c	Ordinary two-way ANOVA with Tukey's multiple comparisons			
IL-1b	-	*Nlrp3*$^{+/+}$ vs. *Nlrp3*$^{+/-}$	0.9997	ns
		Nlrp3$^{+/+}$ vs. *Nlrp3*$^{-/-}$	0.9992	ns
		Nlrp3$^{+/+}$ vs. *Nlrp3*$^{S803D/+}$	0.9855	ns
		Nlrp3$^{+/+}$ vs. *Nlrp3*$^{S803D/S803D}$	0.9811	ns

	LPS	*Nlrp3*$^{+/+}$ vs. *Nlrp3*$^{+/-}$	0.9954	ns
		Nlrp3$^{+/+}$ vs. *Nlrp3*$^{-/-}$	0.9999	ns
		Nlrp3$^{+/+}$ vs. *Nlrp3*$^{S803D/+}$	0.9989	ns
		Nlrp3$^{+/+}$ vs. *Nlrp3*$^{S803D/S803D}$	0.9862	ns
	LPS+Nig	*Nlrp3*$^{+/+}$ vs. *Nlrp3*$^{+/-}$	<0.0001	****
		Nlrp3$^{+/+}$ vs. *Nlrp3*$^{-/-}$	<0.0001	****
		Nlrp3$^{+/+}$ vs. *Nlrp3*$^{S803D/+}$	<0.0001	****
		Nlrp3$^{+/+}$ vs. *Nlrp3*$^{S803D/S803D}$	<0.0001	****
IL-18	-	*Nlrp3*$^{+/+}$ vs. *Nlrp3*$^{+/-}$	0.9973	ns
		Nlrp3$^{+/+}$ vs. *Nlrp3*$^{-/-}$	0.8711	ns
		Nlrp3$^{+/+}$ vs. *Nlrp3*$^{S803D/+}$	0.0958	ns
		Nlrp3$^{+/+}$ vs. *Nlrp3*$^{S803D/S803D}$	0.9509	ns
	LPS	*Nlrp3*$^{+/+}$ vs. *Nlrp3*$^{+/-}$	0.9962	ns
		Nlrp3$^{+/+}$ vs. *Nlrp3*$^{-/-}$	0.9976	ns
		Nlrp3$^{+/+}$ vs. *Nlrp3*$^{S803D/+}$	0.6729	ns
		Nlrp3$^{+/+}$ vs. *Nlrp3*$^{S803D/S803D}$	0.9999	ns
	LPS+Nig	*Nlrp3*$^{+/+}$ vs. *Nlrp3*$^{+/-}$	<0.0001	****
		Nlrp3$^{+/+}$ vs. *Nlrp3*$^{-/-}$	<0.0001	****
		Nlrp3$^{+/+}$ vs. *Nlrp3*$^{S803D/+}$	<0.0001	****
		Nlrp3$^{+/+}$ vs. *Nlrp3*$^{S803D/S803D}$	<0.0001	****
Sup Fig. 4f	Ordinary two-way ANOVA with Tukey's multiple comparisons			
IL-1b	-	*Nlrp3*$^{+/+}$ vs. *Nlrp3*$^{-/-}$	0.9996	ns
		Nlrp3$^{+/+}$ vs. *Nlrp3*$^{S803D/+}$	>0.9999	ns
		Nlrp3$^{+/+}$ vs. *Nlrp3*$^{S803D/S803D}$	0.9989	ns
	Pam3	*Nlrp3*$^{+/+}$ vs. *Nlrp3*$^{-/-}$	0.8053	ns
		Nlrp3$^{+/+}$ vs. *Nlrp3*$^{S803D/+}$	0.9064	ns
		Nlrp3$^{+/+}$ vs. *Nlrp3*$^{S803D/S803D}$	0.7277	ns
	Pam3+tLPS	*Nlrp3*$^{+/+}$ vs. *Nlrp3*$^{-/-}$	<0.0001	****
		Nlrp3$^{+/+}$ vs. *Nlrp3*$^{S803D/+}$	<0.0001	****
		Nlrp3$^{+/+}$ vs. *Nlrp3*$^{S803D/S803D}$	<0.0001	****
Sup Fig. 8	Two-sided Mantel-Cox test			
	LPS	*Nlrp3*$^{+/+}$ vs. *Nlrp3*$^{S803D/S803D}$	0.0342	*

Supplementary Table 5. Statistical tests and P values.
