# Supplementary material for: NLRP3 phosphorylation in its LRR domain critically regulates inflammasome assembly
Source: Nat Commun. 2021 Oct 6;12:5862. doi: 10.1038/s41467-021-26142-w (PMC8494922; doi:10.1038/s41467-021-26142-w)
Supplement: Supplementary file 3 — Reporting Summary [file 41467_2021_26142_MOESM3_ESM.pdf]

nature portfolio

Corresponding author(s): Benedicte Py

Last updated by author(s): Sep 6, 2021

Reporting Summary

Nature Portfolio wishes to improve the reproducibility of the work that we publish. This form provides structure for consistency and transparency in reporting. For further information on Nature Portfolio policies, see our Editorial Policies and the Editorial Policy Checklist.

Statistics

For all statistical analyses, confirm that the following items are present in the figure legend, table legend, main text, or Methods section.

n/a	Confirmed	
☐	☒	The exact sample size (*n*) for each experimental group/condition, given as a discrete number and unit of measurement
☐	☒	A statement on whether measurements were taken from distinct samples or whether the same sample was measured repeatedly
☐	☒	The statistical test(s) used AND whether they are one- or two-sided<br>*Only common tests should be described solely by name; describe more complex techniques in the Methods section.*
☐	☒	A description of all covariates tested
☐	☒	A description of any assumptions or corrections, such as tests of normality and adjustment for multiple comparisons
☐	☒	A full description of the statistical parameters including central tendency (e.g. means) or other basic estimates (e.g. regression coefficient) AND variation (e.g. standard deviation) or associated estimates of uncertainty (e.g. confidence intervals)
☐	☒	For null hypothesis testing, the test statistic (e.g. *F*, *t*, *r*) with confidence intervals, effect sizes, degrees of freedom and *P* value noted<br>*Give P values as exact values whenever suitable.*
☒	☐	For Bayesian analysis, information on the choice of priors and Markov chain Monte Carlo settings
☒	☐	For hierarchical and complex designs, identification of the appropriate level for tests and full reporting of outcomes
☒	☐	Estimates of effect sizes (e.g. Cohen's *d*, Pearson's *r*), indicating how they were calculated

Our web collection on statistics for biologists contains articles on many of the points above.

Software and code

Policy information about availability of computer code

Data collection	Bio-Rad Image Lab (version 6.0.0 build 25), High Content Microscopy CQ1 Yokogawa (version 1.05.01.01), ClustalW2, Bio-Rad Luminex Bioplex manager (version 6.1), Tecan i-control (version 2.0), Bio-Rad CFX Maestro (version 1.1)
Data analysis	Bio-Rad Image Lab manager (version 1.2.0.12), High Content Microscopy CQ1 Yokogawa (version 1.05.01.01), Excel (version 16.52), Prism (version 7.0e), Image J (version 1.43m), PyMOL (version 2.1), PBEQ solver from CHARMM-gui server, high throughput MD software with CHARMM36m force field ACEMD (version 3.2.3.), ligplot+ (version 1.4), Bio-Rad CFX Maestro (version 1.1), GPS (version 3.0), NetPhos (version 2.0), Sequest (version 28 rev 13), Ascore Module modScore (version 3.0).

For manuscripts utilizing custom algorithms or software that are central to the research but not yet described in published literature, software must be made available to editors and reviewers. We strongly encourage code deposition in a community repository (e.g. GitHub). See the Nature Portfolio guidelines for submitting code & software for further information.

Data

Policy information about availability of data

All manuscripts must include a data availability statement. This statement should provide the following information, where applicable:

- Accession codes, unique identifiers, or web links for publicly available datasets
- A description of any restrictions on data availability
- For clinical datasets or third party data, please ensure that the statement adheres to our policy

All data supporting the findings of this study are provided in the Article and its Supplementary Information, or from the corresponding author on reasonable request. Source data are provided with this paper.

This study used data available in the PDB database (6NPY.PDB)

Field-specific reporting

Please select the one below that is the best fit for your research. If you are not sure, read the appropriate sections before making your selection.

☒ Life sciences ☐ Behavioural & social sciences ☐ Ecological, evolutionary & environmental sciences

For a reference copy of the document with all sections, see nature.com/documents/nr-reporting-summary-flat.pdf

Life sciences study design

All studies must disclose on these points even when the disclosure is negative.

Sample size	No sample size calculation was performed and sample sizes were determined empirically.<br>For in vivo experiments, 11 KI and 10 WT mice littermates were used in the survival experiment, and 7 KI and 7 WT littermates were used in serum cytokine measurement. These sample sizes are standard in the field for these assays and previously used by others to show similar effects (ref Kayagaki et al., Science, 2013, DOI: 10.1126/science.1240248, Sutterwala et al., Immunity, 2006, DOI: 10.1016/j.immuni.2006.02.004, Song et al., Mol Cell, 2017, DOI: 10.1016/j.molcel.2017.08.017)<br>For ELISA and LDH assays, experiments were performed in 2-3 biological replicates representative of 2-4 independent experiments. These sample sizes are standard in the field for these assays and previously used by others to show similar effect (ref Song et al., Mol Cell, 2017, DOI: 10.1016/j.molcel.2017.08.017; Dufies et al., Nature Microbiology, 2020, DOI: 10.1038/s41564-020-00832-5; Hafner-Bratkovic et al., Nature Communications, 2018, DOI: 10.1038/s41467-018-07573-4).<br>For microscopy, ASC speck experiments were performed in 10-11 technical replicates representative of 2-4 independent experiments with total number of cells analyzed between 292-408 (fig. 2d) and 1833-2303 (Fig 4f); FLICA stainings were performed in biological triplicates representative of 2 independent experiments (Fig. 4d), with total number of cells analyzed between 656-1005. These sample sizes are standard in the field for these assays and previously used by others to show similar effect (ref Song et al., Mol Cell, 2017, DOI: 10.1016/j.molcel.2017.08.017; Dufies et al., Nature Microbiology, 2020, DOI: 10.1038/s41564-020-00832-5).
Data exclusions	No data were excluded
Replication	Each in vitro experiment was successfully repeated in 2-4 independent experiments. Fig 1a corresponds to the one selected experiment used to perform the mass spectrometry analysis (out of 8 independent repeats). Fig 4b and Supplementary Figs. 2f, 2g, 3c, 3d, 3e, 4 c-l, 7a, 7b were performed as biological duplicates representative of 2 independent experiments. Figs 2a, 3b, 4a and Supplementary Figs. 2b, 2c, 2d, 2e, 3b, 5a were performed as biological duplicates representative of 3 independent experiments. Figs 2b and 3a were performed as biological duplicates representative of 4 independent experiments. Fig 4d was performed as biological triplicates representative of 2 independent experiments. Fig 7b was performed as biological triplicates representative of 4 independent experiments. Figs 4b, 7a, 7c were performed as biological quadruplicates representative of 2 independent experiments. Supplementary Fig. 7c was performed as biological quadruplicates representative of 3 independent experiments. Supplementary Fig. 7d was performed as technical duplicates in 2 independent experiments. Fig. 2d was performed as 10-11 technical replicates representative of 4 independent experiments. Fig. 4f was performed as 10 technical replicates representative of 2 independent experiments. Fig. 7d was performed as 11 technical replicates representative of 2 independent experiments.<br>Figs 2c, 7e, 7h, 7i and Supplementary Figs. 2a, 2b, 2h, 2i, 3a, 3b, 4b, 5d, 5g, 5h, 5j, 7e are representative of 2 independent experiments. Figs 2e, 4c, 4g, 5a-j, 7f, 7g and Supplementary Figs. 5 a, 5c, 5i are representative of 3 independent experiments. Figs 4e are representative of 4 independent experiments.<br>For in vivo data (Fig. 6 and Supplementary Fig. 6), all individuals are represented due to ethic concerns (n>=10 for survival assay, mantel-cox test $p<0.01$, n=7 for serum cytokine measurement, ANOVA test).
Randomization	For animal studies, WT and KI mice are littermates with matched age and sex.<br>For in vitro/in cellulo studies, samples (cells of each genotype) were randomly allocated from one single pool into experimental groups (corresponding to each treatment)
Blinding	For animal studies, WT and KI mice are littermates with matched age and sex. WT and KI mice are therefore in the same cages and cannot be distinguished by the person performing the experiments. Mice are identified by a numbering system within the cage independent of their genotypes.<br>For in vitro/in cellulo, investigators were not blinded to group allocation during data collection and analysis, as the same investigator was doing the group allocation (cell plating and treatment) and the data collection and analysis. To limit biais, all samples were handled in parallel using to a maximum automated pipetting in 96 well plate format (ELISA, LDH assay, PI incorporation assay) or 24 well format (LDH assay), or in anonymously numbered 1.5 ml tubes (lysates, Immunoprecipitation).

Reporting for specific materials, systems and methods

We require information from authors about some types of materials, experimental systems and methods used in many studies. Here, indicate whether each material, system or method listed is relevant to your study. If you are not sure if a list item applies to your research, read the appropriate section before selecting a response.

Materials & experimental systems

n/a	Involved in the study
☐	☒ Antibodies
☐	☒ Eukaryotic cell lines
☒	☐ Palaeontology and archaeology
☐	☒ Animals and other organisms
☒	☐ Human research participants
☒	☐ Clinical data
☒	☐ Dual use research of concern

Methods

n/a	Involved in the study
☒	☐ ChIP-seq
☒	☐ Flow cytometry
☒	☐ MRI-based neuroimaging

Antibodies

Antibodies used

anti-Actin (Sigma-Aldrich, MAB1501, clone C4, lot 3590048), anti-ASC (Adipogen, AG-25B-0006-C100, clone AL177, lot A40922001), anti-ASC N-15 (Santa Cruz Biotechnology, sc-22514-R, lot I0712), anti-BRCC3 (Cell Signaling Technology, 18215S, clone D5E5H, lot 1), anti-Caspase-1 (BioLegend, 645102, clone 5B10, lot B257128), anti-CK1 alpha (Bethyl Laboratories, A301-991A, lot 1), anti-CK1 alpha (Santa Cruz Biotechnology, sc-74582, clone H-7 lot J0620), anti-Flag (Sigma-Aldrich, F3165, clone M2, lot SLCG2330), anti-HA (Sigma-Aldrich, H3663, clone HA-7, lot 092M4827V), anti-IL-1b (R&D Systems, MAB4011, clone 166926), anti-IL-1b (Cell Signaling Technology, 12703, clone D3U3E, lot 2), anti-LaminB1 B-10 (Santa Cruz Biotechnology, sc-374015, lot F1014), anti-Myc (Sigma-Aldrich, C3956), anti-NEK7 (Abcam, ab133514, clone EPR4900, lot GR3258619-1), anti-NLRP3 (Adipogen, AG-20B-0014-C100, clone Cryo2, lot A41812012), anti-pNLRP3S198 (From Dr. Tao Li, Beijing China), anti-Ubiquitin (Dako, Z0458, lot 94251), anti-Ubiquitin (Santa Cruz Biotechnology, sc-8017, lot H2719), anti-Ubiquitin (Cell Signaling Technology, 3933, lot 6), anti-Ubiquitin (K48) (Cell Signaling Technology, 4289, lot 2), anti-Ubiquitin (K63) (Cell Signaling Technology, 5621, lot 5), anti-VSV (Sigma-Aldrich, V5507, clone P5D4, lot 035M4777U), anti-phospho(Ser/Thr) (Cell Signaling Technology, 9631S, lot 10), anti-Mouse IgG (H+ L)-HRP (Promega, W402B, lot 00441155), anti-Rabbit IgG (H+L)-HRP (Promega, W401B, lot 390794), anti-Rat IgM+IgG (H+L)-HRP (Southern Biotech, 3010-05, lot G2512-M748B), anti-Rabbit IgG (H+L)-HRP (Invitrogen, A21074), IgG (Diagenode, C15410206, lot RIG001AM), anti-Flag agarose (Sigma-Aldrich, A22220, clone M2), anti-HA agarose (Sigma-Aldrich, A2095, clone HA-7, lot 048M4893V).
Dilutions of all antibodies for each applications is described in the Methods section and in Supplementary Table 4.

Validation

According to the manufacturers'datasheets, the antibodies are validated for the following applications: anti-Actin, Species Human/Mouse, Application WB (Sigma-Aldrich, MAB1501); anti-ASC, Species Human/Mouse, Application WB/IF/IP (Adipogen, AG-25B-0006-C100), anti-ASC N-15, Species Human/Mouse, Application WB/IF/IP (Santa Cruz Biotechnology, sc-22514-R); anti-BRCC3, Species Human/Mouse, Application WB/IP (Cell Signaling Technology, 18215S); anti-Caspase-1, Species Human/Mouse, Application WB (BioLegend, 645102); anti-CK1 alpha, Species Human/Mouse, Application WB/IP (Bethyl Laboratories, A301-991A); anti-CK1 alpha, Species Human/Mouse, Application WB (Santa Cruz Biotechnology, sc-74582); anti-Flag, Application WB (Sigma-Aldrich, F3165); anti-HA, Application WB (Sigma-Aldrich, H3663); anti-IL-1b, Species Human/Mouse, Application WB (R&D Systems, MAB4011); anti-IL-1b, Species Human/Mouse, Application WB (Cell Signaling Technology, 12703); anti-LaminB1 B-10, Species Human/Mouse, Application WB (Santa Cruz Biotechnology, sc-374015); anti-Myc, Application WB/IP (Sigma-Aldrich, C3956); anti-NEK7, Species Human/Mouse, Application WB/IP (Abcam, ab133514); anti-NLRP3, Species Human/Mouse, Application WB/IP (Adipogen, AG-20B-0014-C100), anti-Ubiquitin, Species Human/Mouse, Application WB (Dako, Z0458); anti-Ubiquitin, Species Human/Mouse, Application WB (Santa Cruz Biotechnology, sc-8017); anti-Ubiquitin, Species Human/Mouse, Application WB (Cell Signaling Technology, 3933); anti-Ubiquitin (K48), Species Human/Mouse, Application WB (Cell Signaling Technology, 4289); anti-Ubiquitin (K63), Species Human/Mouse, Application WB (Cell Signaling Technology, 5621); anti-VSV, Species Human/Mouse, Application WB/IP (Sigma-Aldrich, V5507); anti-phospho(Ser/Thr) (Cell Signaling Technology, 9631S, lot 10), anti-Mouse IgG (H+ L)-HRP (Promega, W402B, lot 00441155), anti-Rabbit IgG (H+L)-HRP, Species Human/Mouse, Application WB (Promega, W401B), anti-Rat IgM+IgG (H+L)-HRP, Species Rat, Application WB (Southern Biotech, 3010-05); anti-Rabbit IgG (H+L)-HRP, Species Rabbit, Application WB (Invitrogen, A21074); anti-Flag agarose, Application IP (Sigma-Aldrich, A22220), anti-HA agarose, Application IP (Sigma-Aldrich, A2095).
Anti-pNLRP3S198 (From Dr. Tao Li, Beijing China) has been validated for Species Human, Application WB (Song et al., Mol Cell, 2017, DOI: 10.1016/j.molcel.2017.08.017).

Eukaryotic cell lines

Policy information about cell lines

Cell line source(s)

HEK-293T, U937 (ATCC); HEK-293T-Lenti-XTM (Takara Bio, 632180); Nlrp3+/+ and Nlrp3-/- Immortalized bone marrow-derived macrophages (iBMDMs) (Pr. Emad Alnemri, Thomas Jefferson University, Philadelphia, USA); NLRP3-/- U937 (Dr Thomas Henry, International Center for Infectiology Research, Lyon, France); Nek7+/+ and Nek7-/- iBMDMs (Pr Gabriel Nunez, University of Michigan, USA).

Authentication

Expression of NLRP3 was checked by WB for U937 vs NLRP3-deficient U937, and Nlrp3+/+ vs Nlrp3-/- iBMDMs. Expression of NEK7 was checked by WB for Nek7+/+ vs Nek7-/- iBMDMs. No additional authentification procedure was performed.

Mycoplasma contamination

All cell lines tested negative for mycoplasma contamination.

Commonly misidentified lines
(See ICLAC register)

No commonly misidentified lines.

Animals and other organisms

Policy information about studies involving animals; ARRIVE guidelines recommended for reporting animal research

Laboratory animals	Nlrp3-/- C57Bl/6J mouse, 7- week old, male; Nlrp3 S803D/S803D C57Bl/6J mouse, 7- week old, male; Nlrp3+/+ C57Bl/6J mouse, 7- week old, male; Brcc3-/- C57Bl/6J mouse, 7- week old, male. Mice were housed at the PBES rodent facility (ENS Lyon, France) under 13h light/11h dark cycle, 22°C +/-2°C, 50% +/-10% humidity.
Wild animals	The study did not involve wild animals.
Field-collected samples	The study did not involve sample collected from the field.
Ethics oversight	The CECCAPP ethic committee and the French Ministry for Superior Education Research and Innovation approved the protocols used in the study.

Note that full information on the approval of the study protocol must also be provided in the manuscript.
